# Comparison of two different labial salivary gland biopsy incision techniques: A randomized clinical trial

**DOI:** 10.4317/medoral.19033

**Published:** 2013-05-31

**Authors:** Alp Saruhano?lu, Mert Ati775 i?kler, Sertan Ergun, Duygu Ofluo?lu, Hakk? Tanyeri775 i?

**Affiliations:** 1 Istanbul University, Faculty of Dentistry, Department of Oral and Maxillofacial Surgery; 2 Istanbul University, Faculty of Dentistry, Department of Oral and Maxillofacial Surgery; 3 Istanbul University, Faculty of Dentistry, Department of Oral and Maxillofacial Surgery; 4 Istanbul University, Faculty of Dentistry, Department of Oral and Maxillofacial Surgery

## Abstract

Objectives: To compare the reliability of two different labial salivary gland biopsy (LSGB) incision techniques (vertical versus horizontal incision techniques) and to report the related complications and discomfort. Study Design: 163 patients who underwent LSGB were included in this study. Patients were randomly divided as vertical incision group (n=81) and horizontal incision group (n=82). Demographic and clinical information of each patient were recorded. A questionnaire was prepared and applied together with Visual Analog Scale (VAS) on the subjects verbally at the 7th day, postoperatively. Intraoperative, short- term and delayed complications were evaluated. Results: The mean age of patients (117 female, 46 male) was 47.3 years (range 19-79 years). Vertical incision technique was associated with less pain (p<0.001), less swelling (p<0.05), less scar formation (p<0.05) and less difficulty in eating (p<0.05) when compared with horizontal incision technique. No statistically significant differences were observed between the 2 groups in terms of hematoma, parasthesia and speech difficulty (p>0.05). Additionally, two subjects in the horizontal incision group revealed permanent paresthesia during the follow-up period of two years. Conclusions: This prospective study demonstrated that the subjects in the vertical incision group had less complication rates and discomfort after labial salivary gland procedure than those in the horizontal incision group.

** Key words:**Salivary gland, biopsy, incision.

## Introduction

Salivary gland biopsy is a simple surgical procedure that is currently extensively used for the diagnosis of Sjogren Syndrome (SS), sarcoidosis, amyloidosis, and other connective infiltrative diseases (1,2). This procedure can be performed in any region where salivary glands are present. Lip is the most commonly selected area reported in the literature because of the presence of the large number of salivary glands, ease of accessibility, ease of anesthesia and lack of major structures to damage (3,4). Labial salivary gland biopsy (LSGB) is a simple surgical procedure but still it can not be accepted as totally atraumatic (5). Complications reported after LSGB are bleeding, external hematoma, swelling, pain and paresthesia (2,4,5).

There are several methods of labial salivary gland biopsy related to the choice of the surgical technique reported in the literature, however there is no universally accepted consenseus criteria established. Six different types of surgical intervention of LSGB such as horizontal incisions, vertical incisions, wedge-shaped incisions, x marked incision, unique small incision tecniques and punch biopsy are defined in the literature (3,5-14). The aim of this prospective study was to compare the reliability of two different LSGB incision techniques (vertical versus horizontal incision techniques) and to evaluate the related complications and discomfort.

## Patient and Methods

252 labial salivary gland biopsies were performed between August 2009 and March 2012 at Istanbul University, Faculty of Dentistry, Department of Oral and Maxillofacial Surgery, as part of an evaluation for primary Sjogren syndrome and sarcoidosis in patients who were referred from the Departments of Rheumatology and Pulmonology. Patients were randomly divided into two groups according to repeated fair coin-tossing and underwent one of two incision techniques for LSGB: horizontal incision, and vertical incision. Out of this patient population, 163 LSGB procedures were included of whereas 89 patients were dropped-out due to some exclusion criteria as follows: being under any kind of medication during the 2 week-period before the procedure, a history of bleeding disorder and presence of pregnancy. This study was approved by the Ethical Comitee of Istanbul University, Istanbul Faculty of Medicine, No: 1983-853.

**Figure 1:**
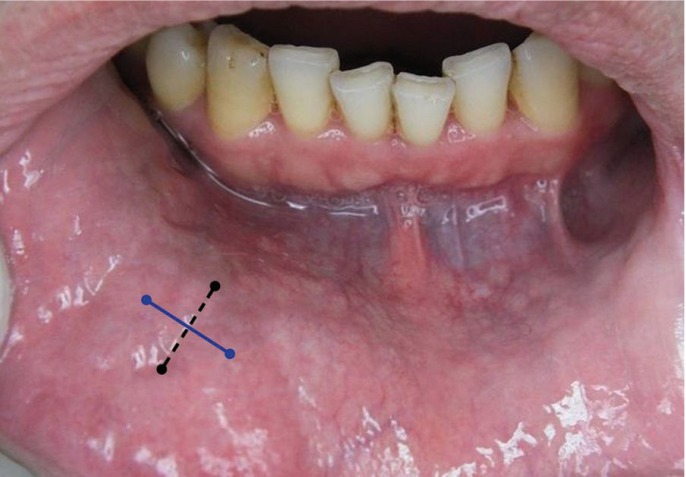
Vertical incision (dotted black) and horizontal incision (straight blue).

All patients provided written informed consent before the procedure and biopsies were performed by the same surgeon under local anesthesia (1ml Lidocaine®, Lidocaine HCL 20mg/ml, Epinephrine HCI 0.0125 mg/ml). Two fingers with sterile sponge were used to hold the lip firmly ensuring haemostasis and lip stabilization. A straight incision (10 mm) was performed with a scalpel (blade 15) on the inner surface of the lower lip. After the incison, glands overhanging from the incision area were easily collected with a forceps. Five glands were excised for histopathologic examination. All incisions were primarily closed with 3 3/0 silk sutures. Sutures were removed on the 7th postoperative day. Vertical incision was performed through the mucous membrane into the subjacent submucosa, between midline and commissure. Incision size was 10 mm straight incision (Fig. 1). Horizontal incision was located on the dorsal aspect of the lower lip parallel to the vermillion border, between midline and commissure, horizontally. Straight incisions were made with a length of 10 mm (Fig. 1).

Patients were examined at the 7th day postoperative period verbally and clinically by 2 clinicians other than the surgeon who performed the labial salivary gland biopsies. A questionnaire was used to report the postoperative symptoms and complications of each patients ( Table 1). To evaluate pain, a visual analog scale (VAS) consisting of a 100 mm horizontal line between the poles of “no pain at all” to “worst pain” was used. Subjects were told to mark the line with a vertical mark at the point that best represented the overall postoperative pain level in 7 days. Hematoma, swelling and scar formation were also examined clinically. The patients who experienced any numbness of the lip evaluated by clinicians (two-point discrimination test) followed up monthly for 2 years till the numbness disappeared.

**Table 1:**
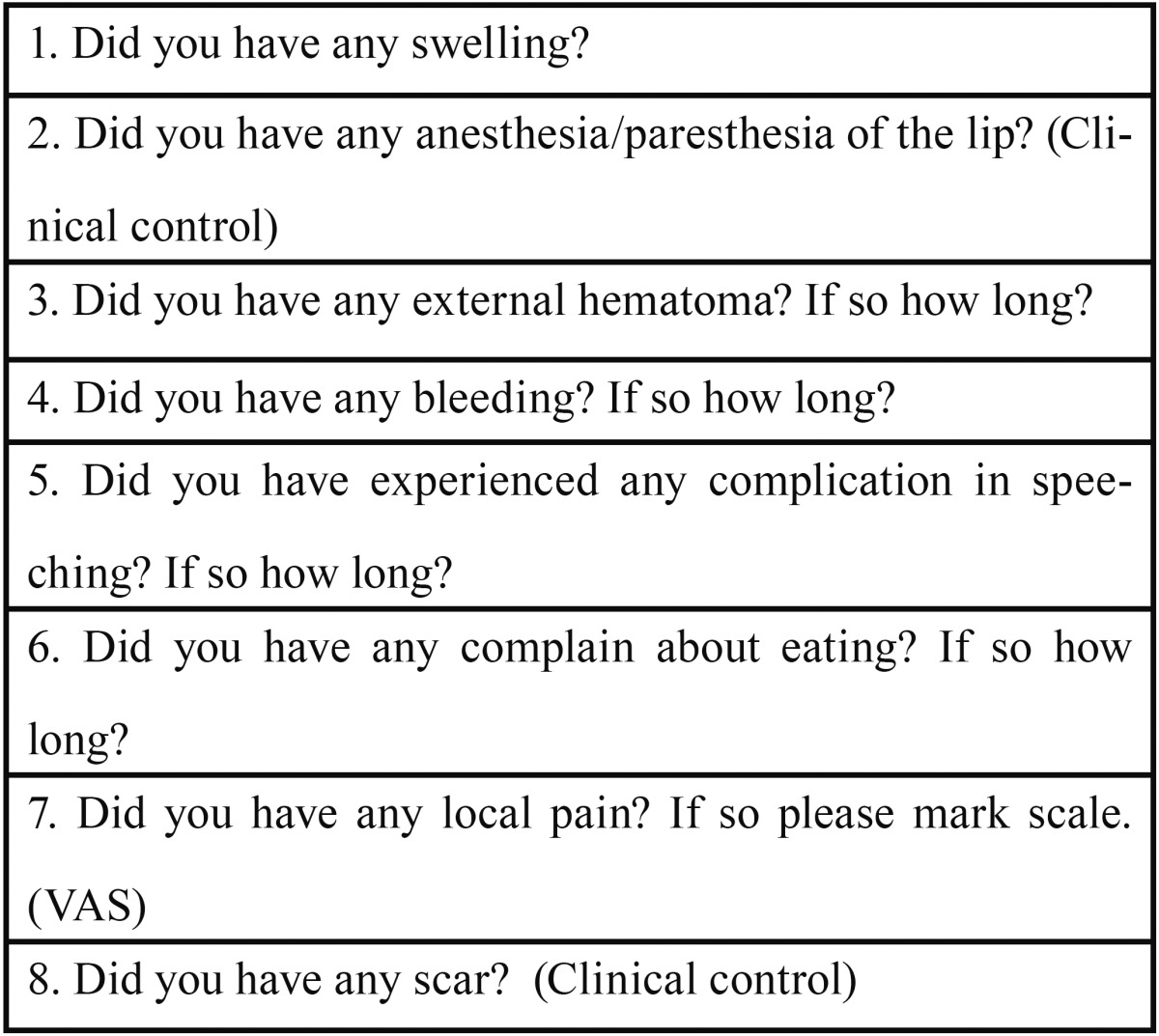
Questionare used to asses the postoperative complications.

Under the assumption of a difference of one standard deviation with respect to the primary end point between the groups, an alpha level 0.01 and the power goal of 90%, minimum 71 patients were necessary; in the present study 81 patients were included in vertical incision group and 82 patients in horizontal group. Number Cruncher Statistical System (NCSS) 2007 & Power Analysis and Sample Size (PASS) 2008 Statistical Software (Utah, USA) programs were used for the statistical analysis. Student-t test was used to evaluate the data regarding the comparisons of descriptive statistical methods (mean, standard deviation, median, frequency, rate). Mann Whitney U test was used for the comparisons of pain scores. Chi-square test and Fisher’s Exact test was used for comparison of qualitative data. Significance was evaluated at a level of p<0.05.

## Results

163 patients (46 male and 117 female) with the mean age of 47.28±12.8 years (range 19-79 years) were included. Labial salivary gland biopsies were performed as part of the evaluation of Sjogren syndrome (n=89); and sarcoidosis (n=74). Out of 163 patients, 82 patients were included in HIG whereas 81 patients were included in VIG. No statistically significant differences were observed between HIG and VIG in terms of age and gender (p>0.05). In each biopsy, adequate number of salivary glands were collected for histopathological examination.

The procedure was well tolerated in all cases with no development of major complications. No statistically significant differences were observed between patients who underwent LSGB for suspected SS versus suspected sarcoidosis (p>0.05). VIG showed statistically significant lower scores in the parameters of pain, swelling, scar formation and difficulty in eating when compared with HIG (p<0.05). No statistically significant differences were found between 2 study groups in terms of hematoma, parasthesia and speech difficulty (p>0.05). None of the patients had any postoperative bleeding. VIG had less VAS scores when compared with those HIG (p<0.001) and the mean VAS scores were 2.90 and 4.28, respectively ( Table 2).

**Table 2:**
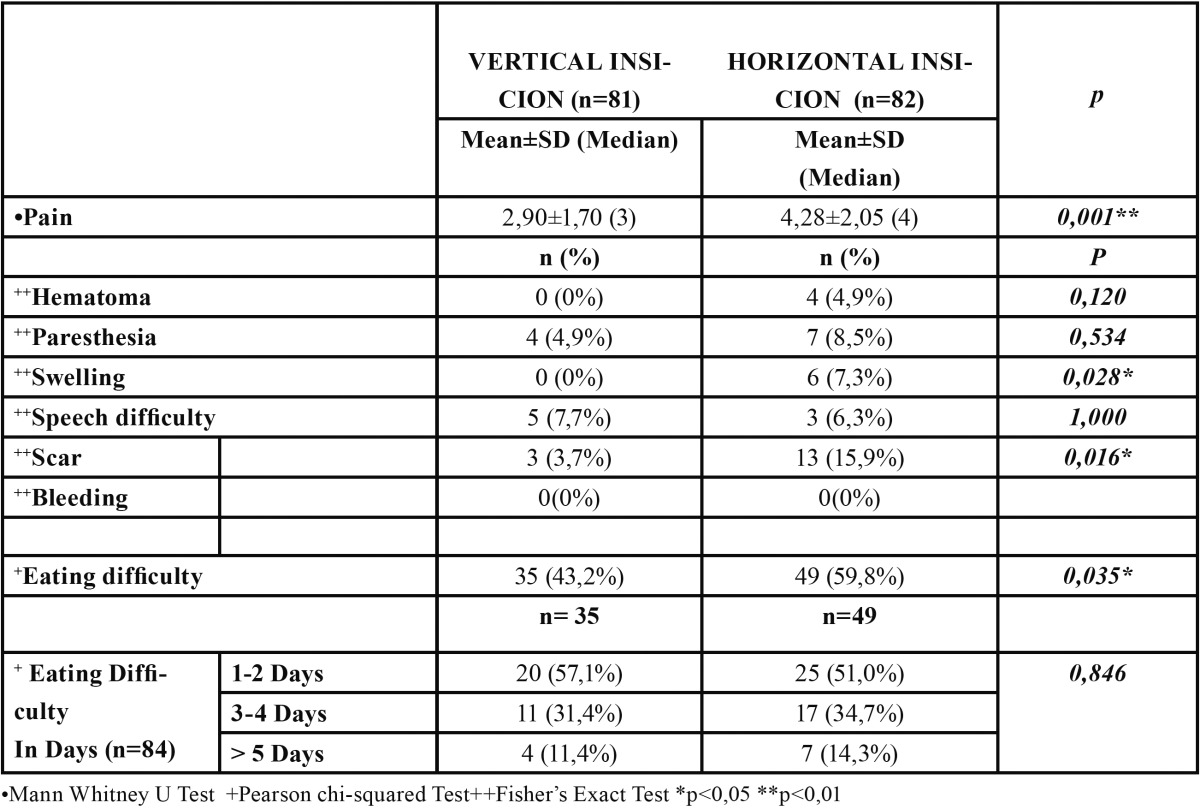
Comparison of vertical incison vs horizontal incision according to complications.

Eight patients (4.90 %, 3 subjects in HIG, 5 subjects in VIG) suffered from difficulty in speech on the same day of the procedure. 84 patients (51.53%, 49 subjects in HIG, 35 subjects in VIG) reported difficulty in eating postoperatively. Out of 84 patients, 45 patients (25 subjects in HIG, 20 subjects in VIG) had eating difficulty in the first 48 hours postoperatively, whereas 39 patients (24 subjects in HIG, 15 subjects in VIG) experienced difficulties in eating up to 5 days.

Eleven patients (6.74 %, 7 subjects in HIG, 4 subjects in VIG) had paresthesia around the incision area, postoperatively. Out of 11 patients, parasthesia disappeared in 5 subjects (4 subjects in HIG, 1 subject in VIG) within 2 months postoperatively whereas in 4 subjects (1 subject in HIG, 3 subjects in VIG) parasthesia disappeared within 2-12 months postoperatively. In 2 years follow-up period, persistent paresthesia was observed in 2 patients (both in HIG).

## Discussion

Labial salivary gland biopsy is a simple, safe and reliable surgical procedure to diagnose glandular involvement in connective-tissue disorders. The procedure was first described by *Chrisholm* et al. in 1963 (15). Different LSGB surgical techniques were reported in the literature since then. Still there is no universally accepted consensus criteria of LSGB reported in the literature.

A review of the literature revealed that the most commonly selected area for LSGB is lateral to the midline, between midline and commissure. The same region was choosen for biopsy in the present study.(1-3,5-9,15-17). The size of the incision was reported in the range of 2-30 mm in length in previous studies (4). Some authors suggested that shorter incisions are more advantageous (2,6,18) because of less complications, whereas others suggested LSGB’s with longer incision techniques to supply sufficient tissue for exact diagnosis (3,15). Teppo and Revonta reported a minimally invasive method in which 3-4 mm horizontal incisions were performed. Among the 191 biopsies that they performed, 3 (1.6%) were considered to have failed due to scarce or non-existent salivary gland tissues in the specimens (18). We performed straight 10 mm incisions in the present study due to our previous clinical experiences which showed that LSGBs with 10 mm incision length revealed less complication rates and adequate number of samples.

As regards the shape of the incision, some authors performed horizontal incisions (3,5,9-11), some performed wedge-shaped incisions (12,13), whereas *Garson* et al. and *Smith* et al. performed vertical insicions (7,8). There were also different incision techniques described in the literature such as the x mark insicion technique by *Peloro* et al. (6), *Caporoli* et al. suggested a unique small incision technique (2) and *Guevera-Gutierrez* et al. suggested punch biopsy to perform LSGB (14). In the present study, we performed horizontal and vertical incision techniques according to our previous experience on LSGB and resembling recent studies of Alantar et al and Alsaad et al (19,20).

The effectiveness of LSGB in the diagnosis of the connective tissue disorders was ruled out in the present study as most related previous studies focused on this topic (10,13,21,22).

The complications reported in the literature were mostly paresthesia around the incision area and/or lip. Only a few studies reported surgical complications such as pyogenic granuloma formation in the area of the incision, external hematoma, local swelling, local infection, internal scarring, local pain, minor bleeding as well as vagal reaction (1,2,5,16,18).

There is only one study by Pijpe et al which reported pain levels of patients who underwent LSGB. Pijpe reported that 31.1% of the patients experienced pain and the mean VAS values were calculated as 7.5 (16). In the present study, out of 163 patients, only 8 subjects did not experience any postoperative pain. This study also revealed that vertical incision caused less pain when compared with the horizontal incision. This can be due to the relationship between the length and localization of the incision as well as the position and direction of the mental nerve together with its branches and the shape.

In the literature, there is lack of information about the difficulty in eating and speaking following after LSGB. The present study could give an opinion to the clinicians that more than half of the patients complained about the eating difficulty postoperatively. Only 8 patients complained about speech difficulty after the operation and all of them complained of difficulty in speaking for one day. None of the patients complained of discomfort in speaking the day after the procedure. This information cannot be compared with the results of the previous studies in this field as there is no reported study which examined these parameters.

A literature review revealed that the most common complication of labial salivary gland biopsies is paresthesia. Out of 16 studies which reported complications of LSGB, 11 revealed parasthesia at least in one case. Caporali et al reported the highest percentage rate of the parasthesia in the literature as %11.35. They also reported that, out of 57 patients with parasthesia postoperatively, only one patient (0.2%) had persistent parasthesia (2). In our study there were 11 patients who suffered from parasthesia and 2 (1.23%) of them revealed still parasthesia in the second year of follow-up which is in accordance with the results of the previous studies (5,7,9,12,14). Alsaad et al. and Alantar et al. evaluated the correlation between the incision types and the position of the mental nerve in cadavers (19,20). Alsaad et al. suggested an oblique incision through the mucous membrane, 1.5 cm lateral to the midline in an inferior-lateral direction, where as Alantar et al suggested horizontal-oblique incision technique carried out across the long axis of the lip with an angle of approximately 36° (19,20). Our study results showed that vertical incision in LSGB seems more reliable than the horizontal incision. This could be related to the relationship between the incisal side of the upper incisors with the incision area where horizontal incision is more affected compared with the vertical incision. Additionally, there is no correlation between the direction of the muscle fibers of orbicularis oris with the direction of the incision as both types of incisions are superficial. One other point was that the affected area was localized between the upper border of the lower lip and the incision line in both cases with permanent parasthesia.

One of the limitation of the study is the evaluation time of the complications. The patients answered the questionare at 7th postoperative day of the procedure. Short term complications (except scar formation) were the common complications during the postoperative period. Thus for further studies diary or daily telephone calls can be more appropriate to compare such complications. One other limitation of the present study is the assesment time of the scar formation. The scar formation evaluated at the 7th day following the operation in the present study, although it is well known that the scar formation can appear later, and it would be better to assess it after some months.

Even though labial salivary gland biopsy is a simple and safe surgical procedure, patients may suffer several complications. Several surgical methods have been established up to date but still there is no universally accepted consensus criteria. For the clinicians it is important to know which surgical intervention is most reliable and also awareness of complications is important to minimize the possible risks, that might be experienced in the postoperative period.

## References

[1] Friedman JA, Miller EB, Huszar M (2002). A simple technique for minor salivary gland biopsy appropriate for use by rheumatologists in an outpatient setting. Clin Rheumatol.

[2] Caporali R, Bonacci E, Epis O, Bobbio-Pallavicini F, Morbini P, Montecucco C (2008). Safety and usefulness of minor salivary gland biopsy: retrospective analysis of 502 procedures performed at a single center. Arthritis Rheum.

[3] Fox PC (1985). Simplified biopsy technique for labial minor salivary glands. Plast Reconstr Surg.

[4] Colella G, Cannavale R, Vicidomini A, Itro A (2010). Salivary gland biopsy: a comprehensive review of techniques and related complications. Rheumatology.

[5] Richards A, Mutlu S, Scully C, Maddison P (1992). Complications associated with labial salivary gland biopsy in the investigation of connective tissue disorders. Ann Rheum Dis.

[6] Peloro TM, Ramsey ML, Marks VJ (2001). Surgical pearl: "X" marks the spot for the salivary gland biopsy. J Am Acad Dermatol.

[7] Gorson KC, Ropper AH (2003). Positive salivary gland biopsy, Sjogren's syndrome and neuropathy: clinical implications. Muscle Nerve.

[8] Smith SR, Shneider BL, Magid M, Martin G, Rothschild M (2004). Minor salivary gland biopsy in neonatal hemochromatosis. Arch Otolaryngol Head Neck Surg.

[9] Daniels TE (1984). Labial salivary gland biopsy in Sjogren's syndrome. Assesment as a diagnostic criterion in 362 suspected cases. Arthritis Rheum.

[10] Greenspan JS, Daniels TE, Talal N, Sylvester RA (1974). The histopathology of Sjogren's syndrome in labial salivary gland biopsies. Oral Surg Oral Med Oral Pathol.

[11] Delgado WA, Mosqueda A (1989). A highly sensetive method for diagnosis of secondary amyloidosis by labial salivary gland biopsy. J Oral Pathol Med.

[12] Berquin K, Mahy P, Weynand B, Reychler H (2006). Accessory or sublingual salivary gland biopsy to assess systemic disease: a comparative retrospective study. Eur Arch Otorhinolaryngol.

[13] Mahlstedt K, Ussmuller J, Donath K (2002). Value of minor salivary gland biopsy in diagnosing Sjogren's syndrome. J Otolaryngol.

[14] Guevara-Gutierrez E, Tlacuilo-Parra A, Minjares-Padilla LM (2001). Minor salivary gland punch biopsy for evaluation of Sjogren's syndrome. J Clin Rheumatol.

[15] Chisholm DM, Mason DK (1968). Labial salivary gland biopsy in Sjogren's disease. J Clin Pathol.

[16] Pijpe J, Kalk W, van der Wal JE, Vissink A, Kluin PM, Roodenburg JE (2007). Parotid gland biopsy compared with labial biopsy in the diagnosis of patients with primary Sjogren's syndrome. Rheumotology.

[17] Seoane J, Varela-Centelles PI, Diz-Dios P, Romero M (2000). Use of chalazion forceps to ease biopsy of minor salivary glands. Laryngoscope.

[18] Teppo H, Revonta M (2007). A follow-up study of minimally invasive lip biopsy in the diagnosis of Sjogren's syndrome. Clin Rheumatol.

[19] Alsaad K, Lee TC, McCartan B (2003). An anatomical study of the cutaneous branches of the mental nerve. Int J Oral Maxillofac Surg.

[20] Alantar A, Roche Y, Maman L, Carpentier P (2000). The lower labial branches of the mental nerve: anatomic variations and surgical relevance. J Oral Maxillofac Surg.

[21] Blaise P, Fardeau C, Chapelon C, Bodaghi B, Le Hoang P (2011). Minor salivary gland biopsy in diagnosing ocular sarcoidosis. Br J Ophtalmol.

[22] Fox R (2011). The importance of minor salivary gland biopsy in prediction of lymphoma in Sjogren's syndrome: should we be obtaining more information about prognosis from minor salivary samples?. Ann Rheum Dis.

